# Acetylcholine modulates gamma frequency oscillations in the hippocampus by activation of muscarinic M1 receptors

**DOI:** 10.1111/ejn.13582

**Published:** 2017-05-08

**Authors:** Ruth T. Betterton, Lisa M. Broad, Krasimira Tsaneva‐Atanasova, Jack R. Mellor

**Affiliations:** ^1^ Centre for Synaptic Plasticity School of Physiology, Pharmacology and Neuroscience University of Bristol Bristol BS8 1TD UK; ^2^ Eli Lilly & Company Ltd. Windlesham Surrey UK; ^3^ Department of Mathematics College of Engineering, Mathematics and Physical Sciences University of Exeter Exeter EX4 4QF UK

**Keywords:** acetylcholine, gamma oscillations, hippocampus, muscarinic M1 receptors

## Abstract

Modulation of gamma oscillations is important for the processing of information and the disruption of gamma oscillations is a prominent feature of schizophrenia and Alzheimer's disease. Gamma oscillations are generated by the interaction of excitatory and inhibitory neurons where their precise frequency and amplitude are controlled by the balance of excitation and inhibition. Acetylcholine enhances the intrinsic excitability of pyramidal neurons and suppresses both excitatory and inhibitory synaptic transmission, but the net modulatory effect on gamma oscillations is not known. Here, we find that the power, but not frequency, of optogenetically induced gamma oscillations in the CA3 region of mouse hippocampal slices is enhanced by low concentrations of the broad‐spectrum cholinergic agonist carbachol but reduced at higher concentrations. This bidirectional modulation of gamma oscillations is replicated within a mathematical model by neuronal depolarisation, but not by reducing synaptic conductances, mimicking the effects of muscarinic M1 receptor activation. The predicted role for M1 receptors was supported experimentally; bidirectional modulation of gamma oscillations by acetylcholine was replicated by a selective M1 receptor agonist and prevented by genetic deletion of M1 receptors. These results reveal that acetylcholine release in CA3 of the hippocampus modulates gamma oscillation power but not frequency in a bidirectional and dose‐dependent manner by acting primarily through muscarinic M1 receptors.

## Introduction

Gamma oscillations are synchronous network oscillations in the 30–100 Hz range found throughout the neocortex and hippocampus. The entrainment of neuronal activity to this high‐frequency oscillation is thought to be important for the timing of spikes both within and between different brain structures determining the flow of information (Colgin *et al*., 2009; Sohal *et al*., 2009; Ainsworth *et al*., 2012). The occurrence of correlated or coherent activity at specific behavioural time points is a critical feature of attention and sensory processing (Fries *et al*., 2001; Womelsdorf *et al*., 2006) and underlies synaptic plasticity required for the encoding of long‐term memory (Kwag & Paulsen, 2009). Moreover, perturbations of gamma oscillation frequency, power and coherence are found in several cognitive disorders including schizophrenia and Alzheimer's disease (Cho *et al*., 2006; Verret *et al*., 2012; Gonzalez‐Burgos *et al*., 2015) indicating the importance of gamma oscillations for cognitive functions.

Generation of gamma oscillations *in vivo* can be achieved by reciprocally connected populations of excitatory and inhibitory neurons (Penttonen *et al*., 1998; Atallah & Scanziani, 2009; Cardin *et al*., 2009; Sohal *et al*., 2009) (see Fig. 4A). This anatomical arrangement occurs throughout the neocortex and hippocampus where inhibitory fast‐spiking parvalbumin‐positive basket cells (PV BCs) provide feedback inhibition of excitatory pyramidal cells (PCs). In the hippocampus gamma oscillations are commonly observed ‘nested’ on the phase of theta oscillations (Lisman & Jensen, 2013), but *in vitro* gamma oscillations may also be induced by application of glutamatergic or cholinergic agonists (Fisahn *et al*., 1998; Palhalmi *et al*., 2004). Gamma oscillation phase can be entrained by strong excitatory or inhibitory input to the network (Akam *et al*., 2012), which is proposed to be a mechanism for the generation of different frequencies of gamma within the same brain region (Colgin *et al*., 2009; Jadi & Sejnowski, 2014; Lasztoczi & Klausberger, 2014; Schomburg *et al*., 2014). However, in comparison to their generation, much less is known about the mechanisms for modulation of gamma oscillations.

The release of acetylcholine in the neocortex and hippocampus activates muscarinic and nicotinic receptors that regulate the processing of information within these circuits (Hasselmo, 2006; Teles‐Grilo Ruivo & Mellor, 2013). Muscarinic and nicotinic receptors are targeted to specific compartments that enable each subtype to control the function of selective nodes within a circuit. For example, M1 muscarinic receptors are principally located on somatic and dendritic compartments of PCs (Levey *et al*., 1995; Yamasaki *et al*., 2010), with some evidence for expression in interneurons (Cea‐del Rio *et al*., 2010; Yi *et al*., 2014), where they increase excitability by causing the opening of non‐selective cationic channels and inhibiting K^+^ channels such as M channels and SK channels (Madison *et al*., 1987; Fisahn *et al*., 2002; Buchanan *et al*., 2010). Genetic deletion of M1 receptors or pharmacological inhibition of muscarinic receptors disrupts memory (Blokland *et al*., 1992; Anagnostaras *et al*., 2003; Atri *et al*., 2004; Wess, 2004; Green *et al*., 2005), whereas administration of muscarinic receptor agonists or acetylcholinesterase inhibitors in Alzheimer's disease (Bodick *et al*., 1997; McGleenon *et al*., 1999) or muscarinic receptor agonists in cognitively impaired humans (Shekhar *et al*., 2008; Nathan *et al*., 2013) can improve memory. Cholinergic agonists and acetylcholinesterase inhibitors induce gamma oscillations *in vitro* (Fisahn *et al*., 2002; Spencer *et al*., 2010), but their role in modulating pre‐existing gamma oscillations and the cholinergic receptor subtypes involved are less well characterised.

To study the mechanisms underlying the modulation of gamma oscillations we made use of *in vitro* and *in silico* models of gamma oscillations. These systems have previously been used to investigate the mechanisms for the generation of gamma oscillations but have rarely been employed to determine the mechanisms by which they might be modulated. Using optogenetically induced gamma oscillations in hippocampal CA3 and a mathematical network model we show that acetylcholine regulates the power, but not frequency, of gamma oscillations in a bidirectional, dose‐dependent manner which is mediated by activation of muscarinic M1 receptors.

## Materials and methods

### Ethical approval

All experiments were performed in accordance with the UK Animal Scientific Procedures Act (1986) and local guidance from the Home Office Licensing Team at the University of Bristol. The protocol was approved by the Animal Welfare and Ethics Review Board at the University of Bristol.

### Transfection

Fifty‐three wild‐type, male, C57/BL6 mice or muscarinic M1 receptor KO mice (M1 KO, bred on a C57/BL6 background; line 1784, Taconic (Fisahn *et al*., 2002)) were used with experiments interleaved for wild‐type and M1 KOs. p21–24 wild‐type mice or p25–40 M1 KO mice underwent stereotaxic surgery to inject virus into the CA3 region of the hippocampus. Under inhalation anaesthesia (1–3% O_2_, 0.5–2% isofluorane), animals were given stereotaxic injections of rAAV5‐CaMKIIa‐hChR2 (H134R)‐EYFP virus (Virus Vector Core; 0.5 μL of 4 × 10^−12^ T.U./mL) at the following coordinates: (from bregma, in mm) posterior 2.3, lateral 2.2, ventral 2.2 (wild‐type mice) and posterior 2.46–2.80, lateral 2.60–2.85, ventral 2.36–3.00 (M1KO mice, coordinates chosen based on animal age). To assess construct expression, animals were anaesthetised with intraperitoneal injection of sodium pentobarbital (150 mg/kg) and perfused transcardially with 4% paraformaldehyde (Sigma‐Aldrich) at 7, 21 and 35 days after transfection. Sixty‐micrometre‐thick brain sections were taken with the aid of a freezing microtome and mounted with Vectashield medium (Vector Laboratories) containing DAPI allowing the visualisation of cell nuclei alongside ChR2‐YFP expression under an epifluorescence microscope.

### Slice electrophysiology

Acute hippocampal slices were prepared 7–40 days after transfection for initial characterisation and 27–53 days after transfection for all drug concentration comparisons. Animals were decapitated following cervical dislocation, the brain removed and hippocampi dissected in ice‐cold modified ACSF containing (in mm): 252 sucrose, 2.5 KCl, 26 NaHCO_3_, 1 CaCl_2_, 5 MgCl_2_, 1.25 NaH_2_PO_4_ and 10 glucose. Transverse hippocampal slices of 400 μm thickness were cut using a microslicer (Leica VT1200S) and stored in standard ACSF containing (in mm): 119 NaCl, 10 glucose, 26 NaHCO_3_, 2.5 KCl, 1 NaH_2_PO_4_, 2.5 CaCl_2_ and 1.3 MgSO_4_ at room temperature for at least 1 h prior to recording. All solutions were saturated with 95% O_2_, 5% CO_2_.

For recording, slices were transferred to a dual perfusion style submerged chamber for increased metabolic supply (RC‐27L; Warner Instruments). Standard ACSF was perfused at a rate of 8.5 mL/min and recording chamber temperature was maintained at 32–34 °C. Slices were visualised using infrared differential interference‐contrast or fluorescence microscopy and YFP fluorescence excited with a 505‐nm‐wavelength LED. Channelrhodopsin was excited with a 470‐nm‐wavelength LED through the 4 × objective on the microscope. Local field potential (LFP) recordings were made using borosilicate glass pipettes with resistance 3–6 MΩ when filled with standard ACSF. LFPs were recorded with a multiclamp 700A amplifier (Molecular Devices), filtered with a Bessel low‐pass filter at 200 Hz and sampled at 10 kHz using a Micro 1401 data acquisition board (CED). No correction was made for background 50 Hz noise. Recordings were made using Signal2 software (CED) and analysed offline using custom written programs in MATLAB.

### Mathematical model

The model for a single cell (node in the network) was based on the model of Kopell *et al*. (Kopell *et al*., 2010) which uses the Hodgkin–Huxley Eqn (1) :

(1) $$C\frac{dV}{dt}=g_{{}_{Na}}m_{\infty }{\left(V\right)}^{3}h\left(V_{Na}-V\right)+g_{K}n^{4}\left(V_{K}-V\right)+g_{L}\left(V_{L}-V\right)+I_{Km}+I_{syn}+I,$$

(2) $$I_{Km}=g_{Km}k_{Km}\left(V_{Km}-V\right).$$

The membrane potential of both PCs and interneurons is governed by the interaction of sodium (*Na*), potassium (*K*) and leak currents (*L*) as well as an applied current (*I*). Pyramidal cells also contain an additional m‐current potassium conductance modelled as in Nowacki *et al*. (2011) and using *V*_*Km* _= −35 mV: Eqn (2). The applied current takes a variety of forms including step, ramp and sine wave functions. In the model, capacitance density (*C*) is measured in μF/cm^2^, voltage (*V*) in mV and time (*t*) in ms. The variables *g*_*Na*_, *g*_*K*_ and *g*_*L*_ are the maximal ionic conductances (mS/cm^2^). *m*,* h* and *n* are rate functions which determine the gating characteristics of the respective channel and are identical to those given in Kopell *et al*. (2010). The gating variables are different between pyramidal and interneurons and determine their distinct firing properties. Initial model parameters based on Kopell *et al*. (2010) are given in Table 1.

**Table 1:** Model parameters. All parameters were based on those used in Kopell *et al*. (2010). Where changes were made the original values from Kopell *et al*. (2010) are given in bold brackets for comparison. Changes in sodium and potassium conductance and reversal potentials were chosen to produce waveforms closer to those we observed experimentally and were within the ranges used in other similar biophysical modelling studies (Traub *et al*., 1994; Wang & Buzsaki, 1996; Ermentrout & Kopell, 1998)

	Parameters		Value		Unit
			Excitatory cells	Inhibitory cells	
Number of cells		*N*_*pr*_	80		
		*N*_*in*_		20	
	Capacitance	*C*	1	1	μF/cm^2^
Conductances	Sodium	*gNa*	50 **(100)**	35	mS/cm^2^
	Potassium	*gK*	20 **(80)**	9	mS/cm^2^
	Leak	*gL*	0.1	0.1	mS/cm^2^
	M‐current	*gK*_*m*_	0.5		mS/cm^2^
Reversal potentials	Sodium	*V*_*Na*_	50	55	mV
	Potassium	*V*_*K*_	−70 **(**−**100)**	−90	mV
	Leak	*V*_*L*_	−67	−65	mV
	M‐current	*V*_*Km*_	−85		mV
Synaptic conductances	E to E	*g*_*E‐E*_	0.5 **(0)**		mS/cm^2^
	I to E	*g*_*I‐E*_	1.5		mS/cm^2^
	I to I	*g*_*I‐I*_		0.7 **(0.5)**	mS/cm^2^
	E to I	*g*_*E‐I*_		1.5 **(0.5)**	mS/cm^2^
Synaptic reversal potentials		*V*_*rev*_	0	−80	mV
Synaptic time constants	Rise time	*τ*_*R*_	0.1	0.3	ms
	Decay time	*τ*_*D*_	3	9	ms

Cells were synaptically coupled in an all‐to‐all configuration using the following expression:

(3) $$I_{syn}=g_{ij}s_{i}\left(t\right)\left(V_{\mathrm{rev}}-V_{j}\right),$$

where *g*_*ij*_ is the maximal conductance of the synapse, *s*_*i*_ is the gating variable and *V*_rev_ is the reversal potential of the synapse. Finally, there was stochastic external drive applied to the network to simulate noise. A network of 80 PCs and 20 interneurons was used to simulate oscillatory activity in the model system. Fewer neurons reduced the power and therefore reliability of gamma oscillations, whereas more neurons increased simulation time without increasing gamma power. Differential equations were solved in MATLAB using the mid‐point method.

To make comparisons with *in vitro* data, Eqn (4) (Bedard *et al*., 2010) was used to give an approximation of LFP.

(4) $$V_{\mathrm{LFP}}=\frac{R_{e}}{4\pi }\sum_{j}\frac{I_{j}}{r_{j}},$$

where *R*_*e*_ is the extracellular resistivity assumed to be 230 Ω/cm, *I*_*j*_ is the sum of the currents of cell *j* and *r*_*j*_ is taken from a normal distribution representing distance between cell and position where LFP is recorded. A subpopulation of 40 PCs and 10 interneurons was used to calculate LFP.

### Analysis

Fast fourier transform (FFT) of LFP data was performed to generate power spectral densities (PSD) using the chronux toolbox (http://chronux.org, Mitra & Bokil, 2008) in MATLAB and a 5 taper multitaper estimate. In all experiments baseline PSDs with no stimulation were subtracted from data with stimulation input to remove background noise. In every case baseline and stimulation PSDs were calculated from downsampled data (1 kHz) and taken from an average of six 2.5 s frames. Subtracted PSDs were smoothed with a moving average filter with span of 35 data points. Gamma oscillation power was calculated as the integral of the PSD between 30 and 100 Hz and theta oscillation power between 4 and 12 Hz. The peak gamma oscillation frequency was measured at the maximum power between 30 and 100 Hz. Gamma and theta oscillation power was baseline subtracted within each slice experiment.

### Statistics

Data are plotted as mean ± SEM throughout the manuscript. Kolmogorov–Smirnov test showed data were normally distributed. Data for drug concentration comparisons were then analysed by one‐way anova followed by *post hoc* two‐sample *t*‐tests assuming unequal variances using Holm–Bonferroni correction to test the null hypothesis that data were the same as baseline for each pharmacological manipulation. Experimental numbers for statistical analysis were taken as the number of slices used. *Denotes *P* < 0.05, **P < 0.01, ***P < 0.001 and no star denotes *P* > 0.05.

## Results

### Optogenetically induced gamma oscillations in hippocampal slices

Excitation of local excitatory and inhibitory networks has been shown to produce gamma‐frequency oscillations in *in vitro* acute brain slice preparations. In the hippocampus, application of glutamatergic or cholinergic agonists provides excitation that generates gamma oscillations in the 30–50 Hz range (Buhl *et al*., 1998; Fisahn *et al*., 1998; Palhalmi *et al*., 2004). However, these persistent gamma oscillations, although robust and therefore amenable to experimental manipulation, are not observed *in vivo*. More recently, gamma oscillations have been evoked *in vivo* and *in vitro* using transient activation of channelrhodopsin (ChR) to provide excitatory drive to the network. This has been achieved in slices of neocortex or hippocampus by expressing ChR in neurons which are then stimulated by step, ramp or sinusoidal waveforms of light (Adesnik & Scanziani, 2010; Akam *et al*., 2012; Pastoll *et al*., 2013; Butler *et al*., 2016). In particular, the use of an optogenetically driven theta‐frequency sine wave produces theta‐nested high‐frequency gamma comparable to that found *in vivo*. The use of optogenetics to induce gamma oscillations provides a system where the modulation of gamma oscillations by pharmacological activation of cholinergic receptors may be tested. Therefore, we developed an optogenetic system for inducing gamma oscillations in the CA3 region of acute hippocampal slices.

**Figure 1:**
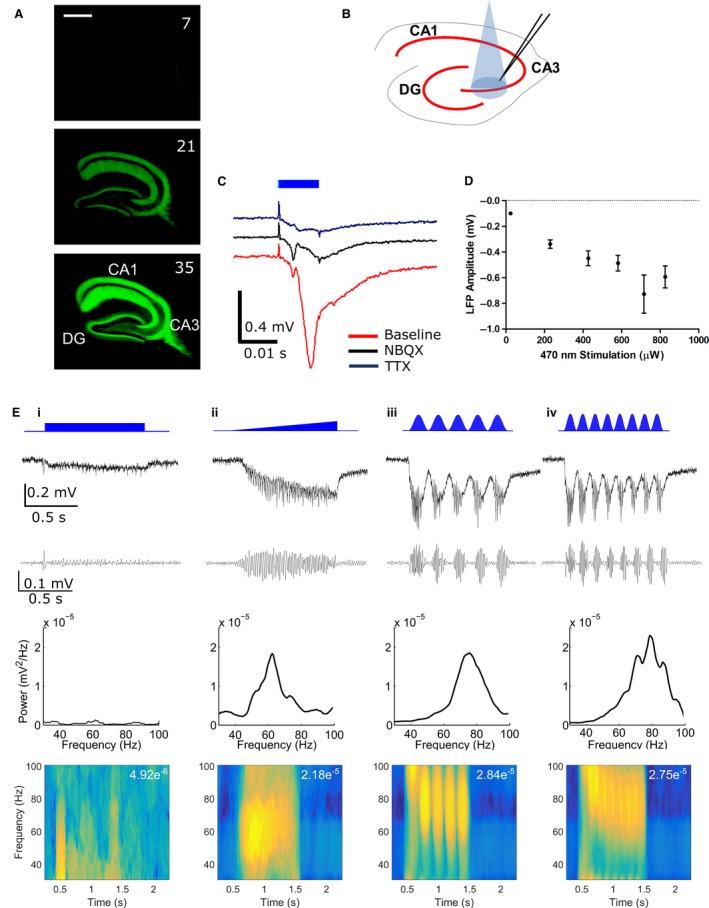
Optogenetic induction of gamma oscillations. (A) Expression of ChR2‐EYFP over time (d.p.i. in top left of each image) after injection of rAAV5‐CaMKIIa‐hChR2 (H134R)‐EYFP virus into CA3 region. Scale bar is 500 μm. (B) Schematic diagram illustrating recording electrode placement within stratum radiatum in CA3 of acute hippocampal slice. (C) Example LFP responses to 10 ms light stimulation (470 nm, 581 μW) during control and following NBQX (10 μm) or TTX (1 μm) application. (D) Amplitude of the LFP response plotted against light intensity (*n* = 10 slices from eight animals). (E) Comparison of optogenetic protocols for induction of gamma oscillations. First row: Schematic of light stimulation protocol. Column i: 1 s step; column ii: 1 s ramp; column iii: 5 Hz sine wave; column iv: 8 Hz sine wave. Subsequent rows show unfiltered LFP, LFP bandpass filtered between 30 and 100 Hz, power spectral density plots and spectrograms (maximum power given in top right). Data shown are from a single slice. [Colour figure can be viewed at wileyonlinelibrary.com].

ChR expression was targeted preferentially to CA3 PCs by stereotaxic injection of a viral vector (AAV5) containing ChR (hChR2 (H134R)) tagged with YFP under the control of the CaMKIIα promoter into the dorsal CA3 region at p21 (see Materials and methods). YFP expression increased over 35 days post‐injection (d.p.i, Fig. 1A) after which expression levels plateaued and appeared strongest in stratum radiatum due to higher membrane density. Functional ChR expression was assessed by LFP recording in stratum radiatum in the CA3 area (Fig. 1B). Ten‐millisecond optical stimulation (470 nm) of a circular area encompassing most of the slice evoked LFP responses comprised of slow (onset > 8 ms after the start of light stimulation) and fast (onset < 5 ms after the start of light stimulation) components which were blocked by NBQX (10 μm) and TTX (1 μm), respectively, and were therefore termed excitatory post‐synaptic potentials (EPSPs) and fibre volleys (Fig. 1C). A residual LFP response was observed in the presence of TTX due to charge flowing through ChR. EPSP amplitude increased with increasing light intensity which plateaued around 600–800 μW (Fig. 1D).

We then tested the relative efficacy of step, ramp or sinusoidal waveforms of light stimulation for the generation of gamma oscillations in hippocampal slices. A representative example of a comparison made in a single slice is shown in Fig. 1E. A 1‐s step waveform (Fig. 1Ei) elicited gamma oscillations with peak frequency 61.5 Hz, but had a low initial power that attenuated rapidly (average power 1.28 × 10^−5^ mV^2^). A ramp stimulus to the same peak light intensity as the step stimulus induced gamma oscillations with similar peak frequency (62.4 Hz) but with higher average power (2.16 × 10^−4^ mV^2^) and less attenuation (Fig. 1Eii). Moreover, gamma oscillation peak frequency did not vary over the course of the ramp stimulation. Hippocampal gamma oscillations *in vivo* are often observed ‘nested’ within an overlying theta (4–12 Hz) oscillation (Bragin *et al*., 1995). We mirrored this in our preparation by stimulating the slice with a 5 Hz or 8 Hz sinusoidal waveform that had the same average light intensity as the step waveform (Fig. 1Eiii and Eiv) (Pastoll *et al*., 2013). These stimulations reliably elicited gamma oscillations of higher peak frequency (75.7 and 78.7 Hz, respectively) and comparable average power (6.80 × 10^−5^ mV^2^ and 8.90 × 10^−5^ mV^2^) with minimal attenuation. Gamma oscillation peak frequency reduced over sequential theta cycles and therefore mean gamma oscillation peak frequency was calculated across the entire 1 s stimulation period (average peak frequency for 5 Hz stimulation was 65.7 ± 3.0 Hz, *n* = 54). In accordance with the YFP expression data (Fig. 1A), expression levels of functional ChR were only sufficient to reliably generate gamma oscillations in response to 5 Hz stimulation around 28 d.p.i. and induced gamma oscillation power was consistent for longer periods of expression post‐injection (average gamma oscillation power 7.98 × 10^−5^ ± 1.27 × 10^−5^ mV^2^, range 27–53 d.p.i., *n* = 54). Given the physiological relevance of theta frequency for the generation of gamma oscillations, the 5 Hz sinusoidal waveform stimulation protocol was selected for all further experiments and the maximum light stimulation intensity was set to the value that elicited a half maximal EPSP response (Fig. 1D).

**Figure 2:**
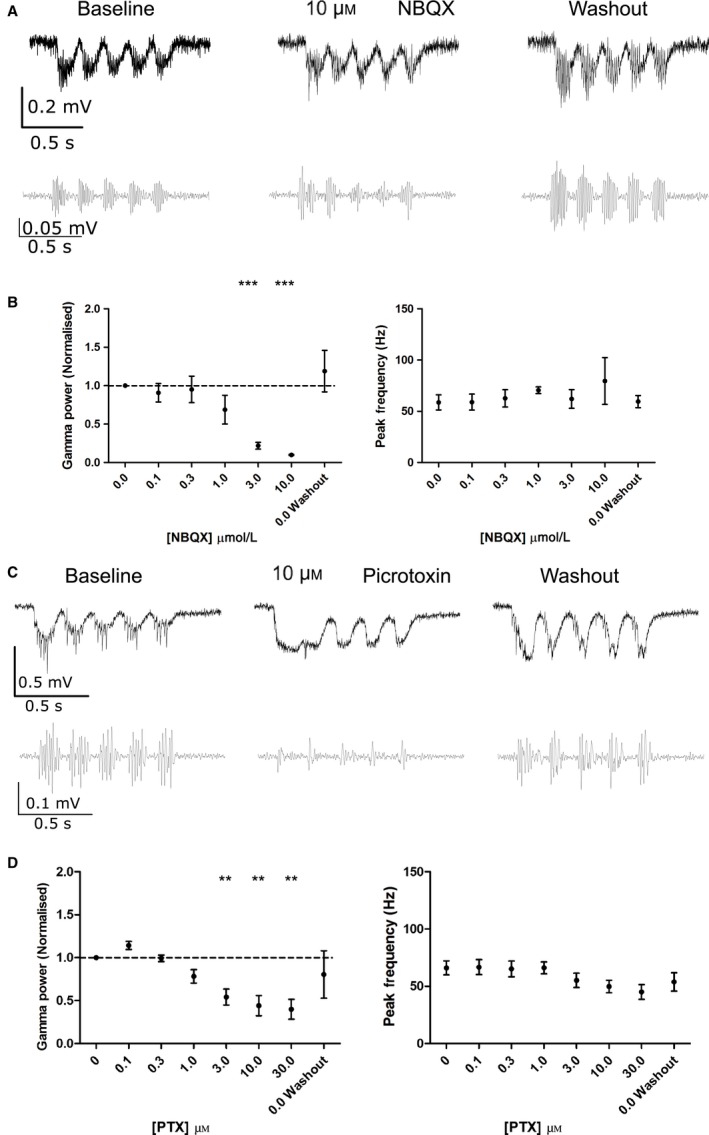
Inhibition of excitatory and inhibitory synaptic connections reduces the power of theta‐nested gamma oscillations. (A) Example unfiltered (top) and bandpass filtered (bottom) LFP traces showing response to 5 Hz sinusoidal light stimulation before, during and after NBQX (10 μm). (B) Gamma oscillation power but not peak frequency was reduced by NBQX at concentrations of 3 and 10 μm (*n* = 9 slices from six animals; *P* = 5.89 × 10^−5^ and 1.92 × 10^−8^ compared to baseline for 3 and 10 μm, respectively, *P* > 0.05 for all other concentrations). (C) Example unfiltered (top) and bandpass filtered (bottom) LFP traces showing response to 5 Hz sinusoidal light stimulation before, during and after picrotoxin (PTX, 10 μm). (D) Gamma oscillation power but not peak frequency was reduced by picrotoxin at concentrations of 3, 10 and 30 μm (*n* = 8 slices from six animals; *P* = 0.00265, 0.00204 and 0.00349 compared to baseline for 3, 10 and 30 μm, respectively, *P* > 0.05 for all other concentrations). **P < 0.01, ***P < 0.001.

In our experimental model, gamma oscillations are hypothesised to be generated by the direct reciprocal interaction between glutamatergic CA3 PCs and GABAergic PV BCs (Adesnik & Scanziani, 2010; Akam *et al*., 2012; Pastoll *et al*., 2013; Butler *et al*., 2016). We tested this by application of glutamatergic or GABAergic antagonists to gamma oscillations induced by 5 Hz, theta‐frequency light stimulation. The AMPA/kainate receptor antagonist NBQX was bath applied at increasing concentrations from 0.1 to 10 μm (Fig. 2A and B). Gamma oscillation power was decreased at concentrations ≥1 μm without affecting peak frequency and was reversed after washout of NBQX. The GABA_A_ receptor antagonist picrotoxin (PTX) also decreased the power of gamma oscillations in a dose‐dependent and reversible manner without affecting peak frequency (Fig. 2C and D). These results support a model where optogenetically induced theta‐nested gamma oscillations are generated by reciprocal excitatory and inhibitory synaptic connectivity within the CA3 network (Buzsaki & Wang, 2012).

### Modulation of optogenetically induced gamma oscillations by carbachol

**Figure 3:**
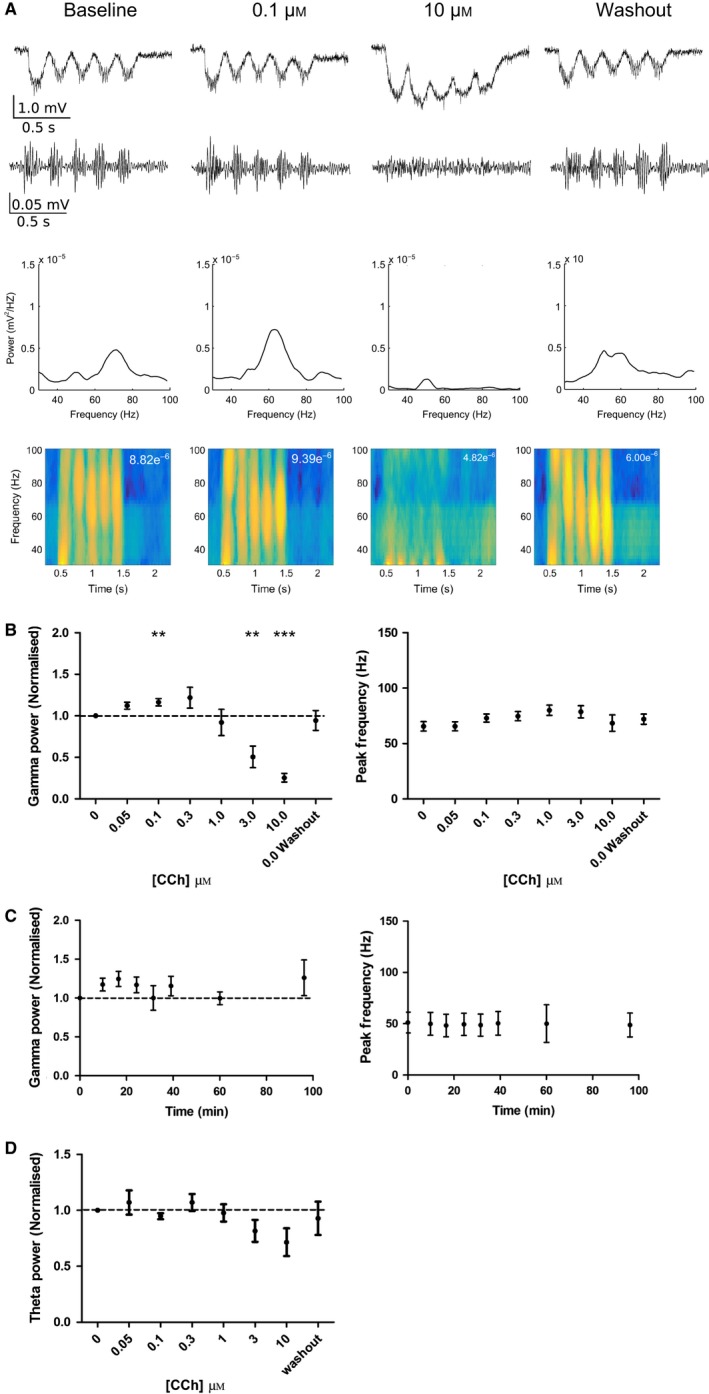
Carbachol induces a dose‐dependent bidirectional change in the power of theta‐nested gamma oscillations. (A) Example unfiltered (top) and bandpass filtered (2nd row) LFP traces showing response to 5 Hz sinusoidal light stimulation before, during and after carbachol (0.1 and 10 μm). Power spectral density plots and spectrograms (maximum power given in top right) are shown below for an example experiment. (B) Gamma oscillation power but not peak frequency was increased at low concentrations (0.1 μm) of carbachol but reduced at higher concentrations (3 and 10 μm) (*n* = 11 slices from nine animals; *P* = 0.00383, 0.00350 and 5.950 × 10^−8^ compared to baseline for 0.1, 3 and 10 μm, respectively, *P* > 0.05 for all other concentrations). (C) Gamma oscillation power and peak frequency were unchanged in time‐matched control experiments (*n* = 4 slices from three animals; *P *> 0.05 for all time points). (D) Theta oscillation power was not changed by any concentration of carbachol (*n* = 11 slices from nine animals; *P* > 0.05 for all concentrations). **P < 0.01, ***P < 0.001. [Colour figure can be viewed at wileyonlinelibrary.com].

We next sought to determine whether acetylcholine could modulate gamma oscillations in our experimental system. The non‐hydrolysable analogue of acetylcholine, carbachol (CCh), was bath applied at increasing concentrations from 50 nm to 10 μm. Low concentrations of CCh (0.05 and 0.1 μm) caused an increase in gamma oscillation power, whereas higher concentrations (3 and 10 μm) caused a decrease in gamma oscillation power (Fig. 3A and B). The peak average frequency of gamma oscillations was not affected by any concentration of CCh (Fig. 3A and B) and neither was the peak frequency during the first theta cycle nor the attenuation of gamma frequency across consecutive theta cycles (Fig. S1). The effects of CCh on the power of gamma oscillations were fully reversed on washout of CCh and, furthermore, time‐matched control experiments showed that gamma oscillation power and peak frequency did not vary over the course of experiments (Fig. 3C). *In vivo* observations indicate that gamma oscillation power is dependent on theta oscillation power (Bragin *et al*., 1995). Therefore, we next analysed whether CCh had any effect on the power of theta oscillations. We found that theta oscillation power remained constant for all concentrations of CCh application (Fig. 3D) indicating that CCh‐induced changes in gamma oscillation power were not attributable to changes in theta oscillation power. In addition, neither theta nor gamma power was increased outside of optogenetic stimulation by these concentrations of carbachol. These results show that CCh induces a bidirectional dose‐dependent effect on the power of gamma oscillations that is independent of underlying theta oscillation power.

### Modelling the modulation of gamma oscillations by acetylcholine

**Figure 4:**
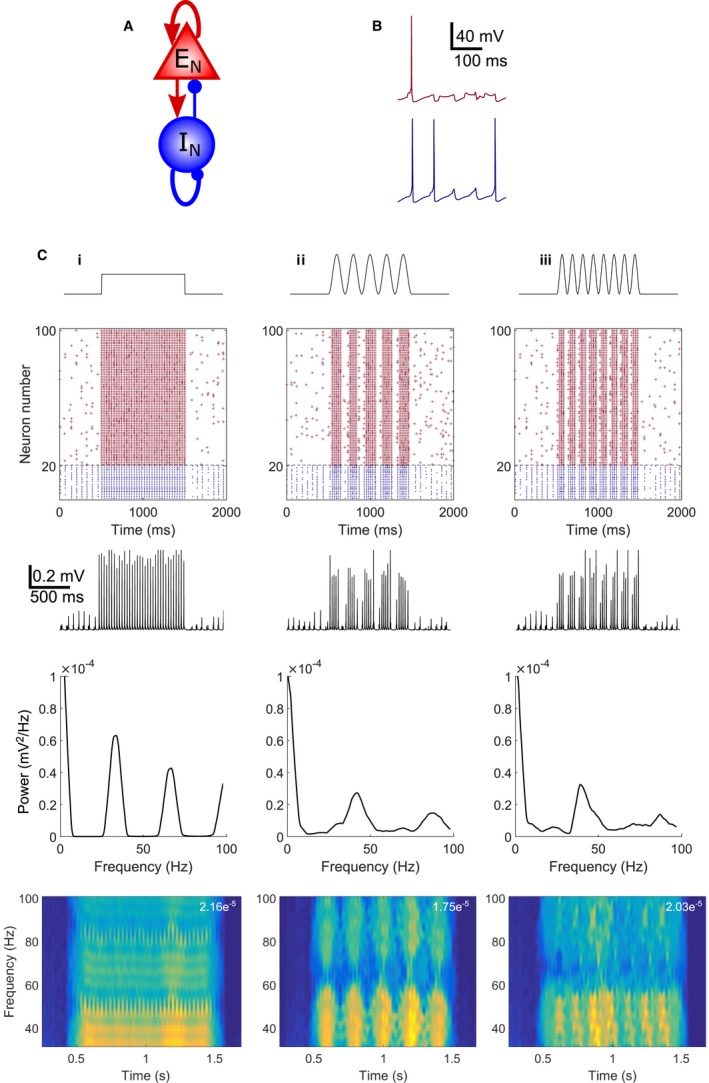
Theta‐nested gamma oscillations in a biophysical network model. (A) Schematic representation for model connectivity between excitatory (E_N_) and inhibitory (I_N_) neurons. (B) Spiking characteristics of excitatory (top, red) and inhibitory (bottom, blue) neurons within the network. (C) Network behaviour in response to step (Ci) or 5 Hz (Cii) or 8 Hz (Ciii) input to excitatory cells with maximal amplitude 2.5 μA/cm^2^. First row: Schematic of current injection protocol; second row: raster plot of spiking for a network of 80 excitatory (red) and 20 inhibitory (blue) neurons; third row: LFP; fourth row: power spectral density plots; fifth row: spectrograms (maximum power given in top right). [Colour figure can be viewed at wileyonlinelibrary.com].

Acetylcholine activates nicotinic α4β2, α3β4 and α7 receptors and muscarinic M1, M2, M3 and M4 receptors in the hippocampus causing a range of effects including inhibition of potassium channels and depolarisation (M1 and M3) and regulation of presynaptic calcium channels and release of neurotransmitter (M2, M4, α4β2, α3β4 and α7) (Teles‐Grilo Ruivo & Mellor, 2013). Acetylcholine and its non‐hydrolysable analogue carbachol have different affinities and efficacies at these cholinergic receptor subtypes (Jensen *et al*., 2003). To investigate the mechanism underlying cholinergic modulation of gamma oscillations and identify which cholinergic receptors are involved we developed a biophysical model of CA3 comprising a network of single‐compartment Hodgkin‐Huxley type neurons, based on the work of Kopell *et al*. (see Materials and methods) (Kopell *et al*., 2010). Eighty PCs and 20 interneurons were all‐to‐all connected (Fig. 4A), fired action potentials (Fig. 4B) and exhibited coordinated network behaviour (Fig. 4C). As previously described in experimental and theoretical studies (Kopell *et al*., 2010; Akam *et al*., 2012), step input to the PCs (1.25 μA/cm^2^) drove the network to fire at gamma frequency (Fig. 4Ci) as did theta‐frequency sinusoidal inputs (2.5 μA/cm^2^ maximum amplitude) at 5 Hz or 8 Hz (Fig. 4Cii, Ciii), thus recapitulating key characteristics of the experimental data. To allow comparisons with our experimental results, we used the 5 Hz sinusoidal input in all further simulations which produced gamma oscillations with peak frequency 41.2 Hz and average power 3.94 × 10^−4^ mV^2^.

**Figure 5:**
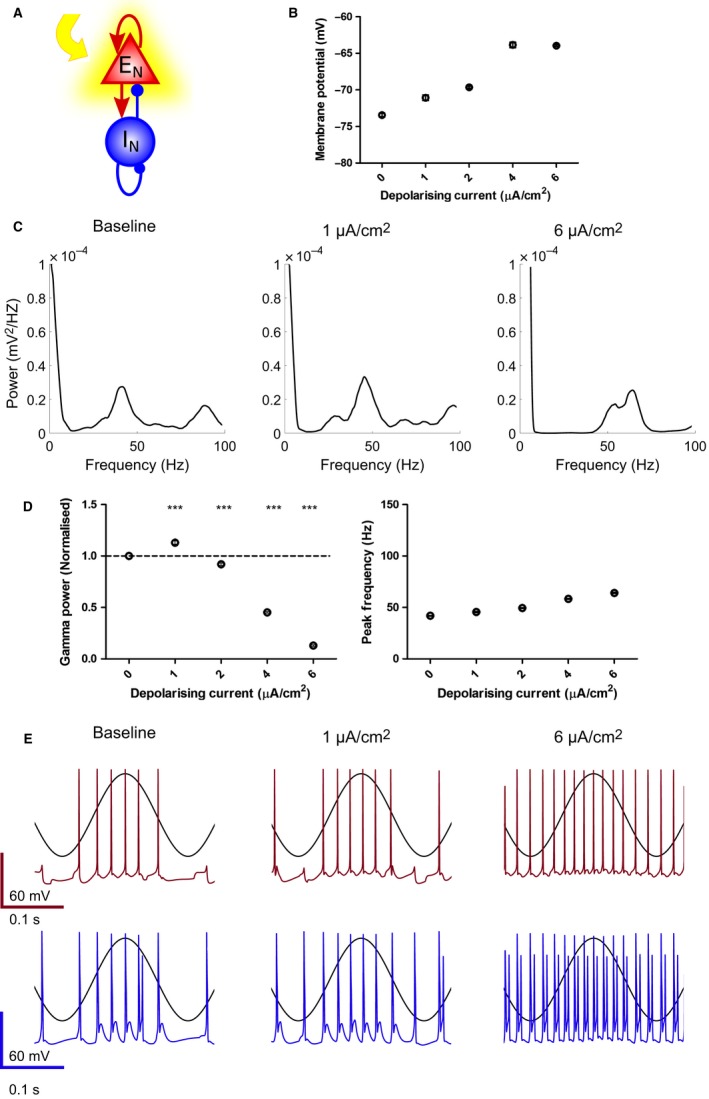
Modelling the effect of M1 mAChR activation on gamma oscillations. (A) Schematic representation of depolarising current given to excitatory neurons within the network to model the action of M1 mAChRs. (B) Increasing the amount of current injection depolarised pyramidal neurons in the mathematical model. (C) Power spectral density plots for increasing current injections. (D) Gamma oscillation power was increased at low current injection (1 μA/cm^2^), but reduced at higher current injections (4 and 6 μA/cm^2^) (*n* = 7; *P* = 2.24 × 10^−4^, 5.09 × 10^−4^, 7.82 × 10^−10^ and 5.56 × 10^−11^ compared to baseline for 1, 2, 4, 6 and 8 μA/cm^2^, respectively). (E) Example spiking output during sinusoidal input to pyramidal cells (black trace overlaid) for excitatory (top) and inhibitory (bottom) neurons over the range of constant current injections given to pyramidal neurons. ***P < 0.001. [Colour figure can be viewed at wileyonlinelibrary.com].

We next tested two hypothesised effects of acetylcholine on gamma oscillations: (i) depolarisation of PCs caused by activation of a non‐selective cationic conductance and inhibition of KCNQ channels (M‐current) which model core aspects of muscarinic M1 receptor activation (Madison *et al*., 1987; Fisahn *et al*., 2002), and (ii) reduction in inhibitory synaptic transmission which models an aspect of muscarinic M2 and nicotinic α4β2, α3β4 and α7 receptor activation (Alkondon & Albuquerque, 2001; Ji *et al*., 2001; Szabo *et al*., 2010; Tang *et al*., 2011; Teles‐Grilo Ruivo & Mellor, 2013). Inhibition of M‐current in the model had no effect on gamma oscillation power or frequency (Fig. S2). To model the effects of activating a non‐selective cationic conductance (which could be voltage or calcium dependent), a constant depolarising input current of increasing amplitude was applied to PCs in addition to the theta‐frequency sinusoidal current injection (Fig. 5A). 1 μA/cm^2^ constant input current caused PCs to depolarise from −72.3 ± 1.4 mV to −69.9 ± 2.1 mV (Fig. 5B) and increased the power of gamma oscillations (Fig. 5C and D). As input current was increased, PCs were more depolarised (to −65.3 ± 0.8 mV at 6 μA/cm^2^) (Fig. 5B) and the power of gamma oscillations was reduced (Fig. 5C and D). The peak gamma oscillation frequency remained relatively unaffected, within a range of input currents, exhibiting only a slight increase (Fig. 5D). We then tested the mechanism for the biphasic effect on gamma oscillation power by analysing the firing properties of neurons during theta‐frequency excitation. 1 μA/cm^2^ input current to PCs caused both PCs and PV BCs to increase their probability of firing across the theta cycle but with the timing of spikes still entrained to gamma frequency (Fig. 5E). However, as the input current was increased up to 6 μA/cm^2^ the entrainment to gamma frequency was lost such that PCs, and therefore PV BCs, fired at much higher frequencies and the network became desynchronised (Gulyas *et al*., 2010; Fig. 5E). These results indicate that activation of M1 mAChRs is sufficient to replicate the experimental data for the biphasic modulation of gamma oscillation power by carbachol and point towards a mechanism involving an increase in non‐selective cation conductance rather than inhibition of M‐current.

**Figure 6:**
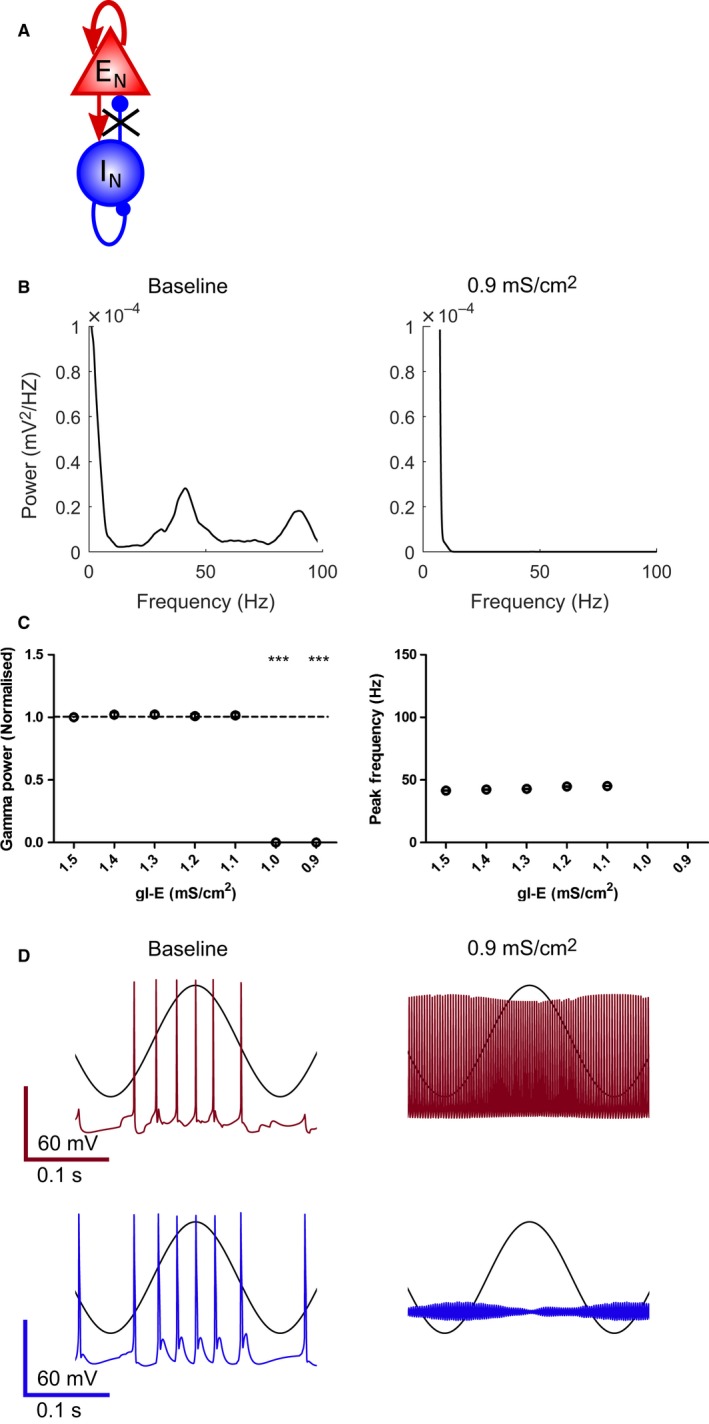
Modelling the effect of M2 mAChR activation on gamma oscillations. (A) Schematic representation of reduction in inhibitory–excitatory synaptic conductance (gI‐E) within the network to model the action of M2 mAChRs. (B) Power spectral density plots for reduced gI‐E. (C) Gamma oscillation power was decreased for reduced gI‐E (1.0 and 0.9 mS/cm^2^) (*n* = 7; *P* = 1.59 × 10^−25^ and 1.37 × 10^−23^ compared to baseline for 1.0 and 0.9 mS/cm^2^, respectively). (D) Example spiking output during sinusoidal input to pyramidal cells (black trace overlaid) for excitatory (top) and inhibitory (bottom) neurons over the range of gI‐E. ***P < 0.001. [Colour figure can be viewed at wileyonlinelibrary.com].

To model the effects of reducing inhibitory synaptic transmission the inhibitory‐to‐excitatory synaptic conductance (gI‐E) was reduced in the model (Fig. 6A). Reducing gI‐E from 1.5 to 0.9 mS/cm^2^ prevented gamma oscillations below a value of 1.1 mS/cm^2^ (Fig. 6B and C). Analysis of neuronal firing during theta‐frequency stimulation revealed that reducing gI‐E below 1.1 mS/cm^2^ caused PV BCs to depolarise sufficiently to inactivate Na^+^ channels and therefore stop firing action potentials leading to a complete loss of rhythmic network activity (Fig. 6D). These results suggest that a critical level of gI‐E is necessary for gamma oscillations, but above this threshold gI‐E does not modulate either their power or frequency.

Overall, these simulations predict that the principal effect of acetylcholine – namely, the bidirectional dose‐dependent increase and decrease in gamma oscillation power – could be explained solely by the activation of M1 mAChRs, although it is possible that the decrease in gamma oscillation power at higher concentrations could also be partly mediated by activation of other cholinergic receptors that depress inhibitory synaptic transmission.

**Figure 7:**
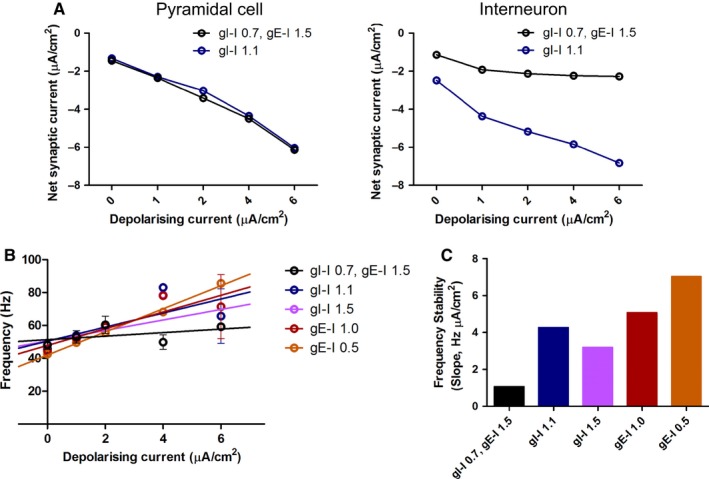
Gamma oscillation frequency stability is governed by synaptic input to interneurons. (A) Net synaptic current density (excitatory synapses are characterised by positive current and vice versa) during modelled gamma oscillations induced by theta‐frequency stimulation became more strongly negative in pyramidal cells (PCs; left) but less so in interneurons (right) as the constant depolarising current increased. Increasing inhibitory‐to‐inhibitory conductance, gI‐I, from 0.7 mS/cm^2^ to 1.1 mS/cm^2^ produced no change in net synaptic current density in PCs, but became more negative in interneurons particularly with large constant depolarising current. (B) Gamma oscillation frequency was less stable across a range of constant current injections when excitatory–inhibitory synaptic balance was reduced. In control conditions (gI‐I = 0.7 mS/cm^2^, gE‐I = 1.5 mS/cm^2^), gamma oscillation frequency increased only slightly with applied depolarising current (fitted plots (left) and slopes (right)). When E‐I balance was altered by either increasing gI‐I or reducing gE‐I gamma oscillation frequency increased strongly with applied depolarising current (*n* = 7; *P* = 8.71 × 10^−3^, 6.77 × 10^−4^, 5.10 × 10^−3^, 2.54 × 10^−8^ for slope comparison control to gI‐I = 1.1 mS/cm^2^, gI‐I = 1.5 mS/cm^2^, gE‐I = 1.0 mS/cm^2^, gE‐I = 0.5 mS/cm^2^, respectively). [Colour figure can be viewed at wileyonlinelibrary.com].

Interestingly, both the experimental and simulation data showed a remarkable stability in gamma oscillation frequency despite considerable modulation of gamma oscillation power across the range of carbachol concentrations (Fig. 3) and current injections (Fig. 5), respectively. The frequency of gamma oscillations has been proposed to be principally governed by the activity of, and therefore synaptic input to, interneurons during ongoing gamma oscillations (Whittington *et al*., 1995; Wang & Buzsaki, 1996; Jadi & Sejnowski, 2014), and therefore we hypothesised that stable gamma oscillation frequency could result from stable synaptic input to interneurons during gamma oscillations. We tested this in the biophysical model by increasing the interneuron‐to‐interneuron synaptic conductance (gI‐I from 0.7 to 1.1 mS/cm^2^), which produced an increase in the net synaptic current (calculated by summating the excitatory and inhibitory synaptic currents during the simulation period) during gamma oscillations in interneurons but not in PCs (Fig. 7A). Furthermore, net synaptic current remained fairly constant across the range of constant current injections to the PCs found to modulate gamma oscillation power when gI‐I = 0.7 mS/cm^2^, but in contrast, the net synaptic current increased substantially when gI‐I = 1.1 mS/cm^2^ as the constant current injection was increased. This suggested that increasing inhibitory synaptic conductance between interneurons reduces the stability of net synaptic current to interneurons during gamma oscillations and therefore the stability of gamma oscillation frequency. Indeed, when we analysed the gamma oscillation frequency stability across the range of depolarising current injections we found that using a low gI‐I produced relatively stable frequencies, but when gI‐I was increased to 1.1 mS/cm^2^ or 1.5 mS/cm^2^ gamma oscillation frequency was much less stable with increasing depolarising current (Fig. 7B and C). Similarly, using an alternative approach to reduce the excitatory–inhibitory synaptic balance to interneurons, we found that reducing excitatory synaptic conductance onto interneurons (gE‐I) from 1.5 mS/cm^2^ to 1.0 mS/cm^2^ or 0.5 mS/cm^2^ also produced much less stable gamma oscillation frequencies (Fig. 7B and C). These results suggest that the gamma oscillation generating network in CA3 of our acute hippocampal slices contains interneurons with relatively large excitatory compared to inhibitory synaptic inputs.

### M1 mAChRs mediate the modulation of gamma oscillations by acetylcholine

To test the model predictions we used a combination of pharmacological and genetic approaches in the experimental optogenetic model of theta‐nested gamma oscillations. The simulations predict that M1 mAChR activation is the principal driver of cholinergic modulation of gamma oscillations, so we tested if M1 mAChR activation was sufficient and necessary using a selective M1 mAChR agonist and M1 mAChR knockout mice.

**Figure 8:**
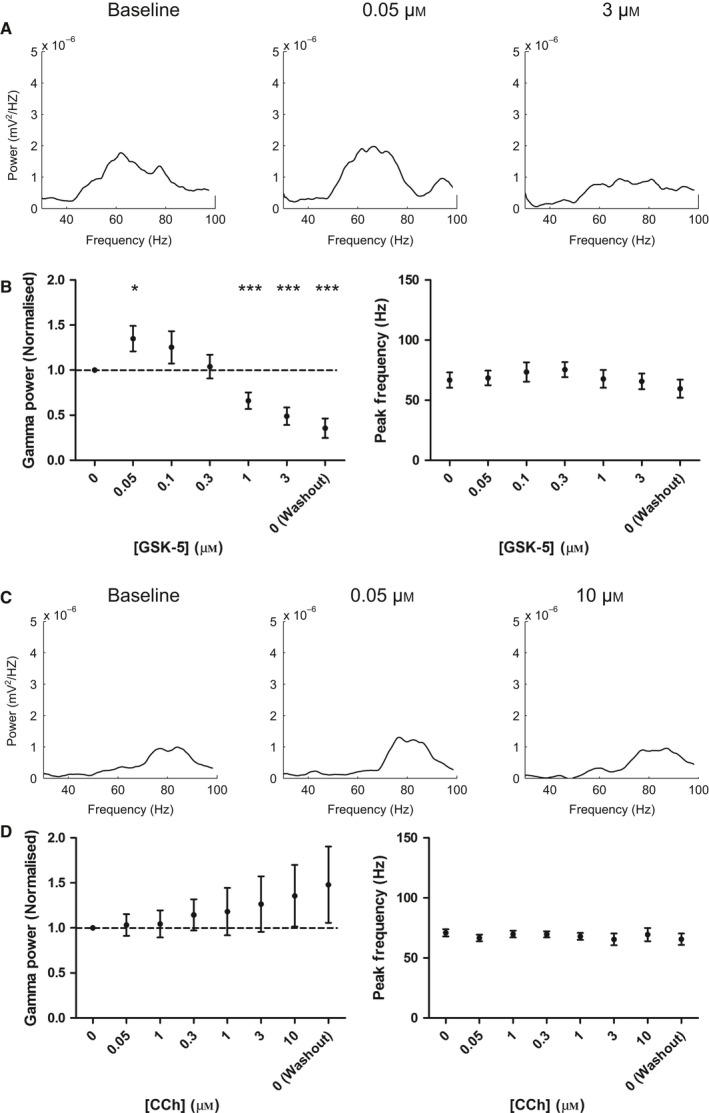
M1 mAChRs are necessary and sufficient for the effects of carbachol on theta‐nested gamma oscillations. (A) Power spectral density plots for theta‐nested gamma oscillations with increasing concentrations of the M1 mAChR selective agonist GSK‐5. (B**)** Gamma oscillation power but not peak frequency was increased at low concentrations of GSK‐5 (0.05 μm), but reduced at higher concentrations (1 and 3 μm) (*n* = 9 slices from six animals; *P* = 0.0164, 5.90 × 10^−3^ and 7.83 × 10^−4^ compared to baseline for 0.05, 1 and 3 μm, respectively, *P* > 0.05 for 0.1 and 0.3 μm;* P* > 0.05 for peak frequency at all concentrations). (C) Power spectral density plots for theta‐nested gamma oscillations with increasing concentrations of carbachol in slices from M1 KO mice. (D) There was no effect of carbachol on gamma oscillation power or peak frequency at any concentration of carbachol (*n *= 9 slices from six animals; *P* > 0.05 for gamma power and peak frequency at all concentrations). *P < 0.05, ***P < 0.001.

The M1 mAChR allosteric agonist GSK‐5 (Budzik *et al*., 2010; Dennis *et al*., 2015) was applied to slices in increasing concentrations from 50 nm to 3 μm. In an almost identical manner to CCh (Fig. 3), GSK‐5 produced a bidirectional dose‐dependent change in the power of gamma oscillations (Fig. 8A and B). Gamma oscillation power increased following applications of 50 nm or 100 nm GSK‐5, whereas 1 μm or 3 μm caused a decrease in power (Fig. 8A and B). We have previously found that GSK‐5 is not readily removed from slices on washout (Dennis *et al*., 2015) and this was also true for the effects on gamma oscillation power. Again, similar to CCh, there was no change in peak gamma oscillation frequency at any concentrations of GSK‐5 tested (Fig. 8B). These results support the mathematical model predictions that M1 mAChRs play a key role in the modulation of gamma oscillations by acetylcholine.

To test whether any other cholinergic receptors are important for the modulation of gamma oscillations, we made use of M1 mAChR knock‐out (M1KO) mice (Fisahn *et al*., 2002; Buchanan *et al*., 2010; Dennis *et al*., 2015). ChR was expressed in the hippocampus following viral injection using the same methods as wild‐type mice and gamma oscillations of similar peak frequency and power were elicited by 5 Hz sinusoidal stimulation. In slices from M1KO mice the effects of CCh application were absent with no increase or decrease in the power or peak frequency of gamma oscillations (Fig. 8C and D). These results strongly indicate that M1 mAChRs are the principal mediators of the bidirectional dose‐dependent effects of acetylcholine on gamma oscillations.

## Discussion

In this study we employed two separate approaches to investigate the modulation of gamma oscillations by acetylcholine. We first developed a model for studying theta‐nested gamma oscillations using theta‐frequency optogenetic stimulation of acute hippocampal slices (Pastoll *et al*., 2013; Butler *et al*., 2016). We found that the broad‐spectrum cholinergic agonist carbachol modulates gamma oscillation power, but not frequency, in a bidirectional and dose‐dependent manner. We then tested the mechanism for this bidirectional modulation using a mathematical biophysical network model for gamma oscillations (Kopell *et al*., 2010) which predicted that M1 mAChRs were the most likely mediators for the effects of acetylcholine. Finally, we tested the model predictions and found that activation of M1 mAChRs is both sufficient and necessary for the modulation of gamma oscillations by acetylcholine.

Theta‐nested gamma oscillations in CA3 region of the hippocampus are generated *in vivo* by the coordinated interactions of excitatory and inhibitory neurons which are triggered by excitation of both groups of neurons phase locked to the theta cycle (Buzsaki & Wang, 2012). This ensures that gamma power strongly covaries with theta power for theta‐nested gamma (Bragin *et al*., 1995). Coordinated excitation of excitatory and inhibitory neurons may also be provided *in vitro* in a persistent manner by different pharmacological mechanisms including stimulation of kainate receptors, metabotropic glutamate receptors or muscarinic receptors (Buhl *et al*., 1998; Fisahn *et al*., 1998; Palhalmi *et al*., 2004). Although gamma oscillations are not thought to be triggered directly by activation of these receptors *in vivo,* this strongly suggests that cholinergic receptor activation can modulate theta‐nested gamma oscillations. The power of theta oscillations in the hippocampus is modulated by cholinergic innervation (Lee *et al*., 1994; Vandecasteele *et al*., 2014) providing one indirect mechanism for the modulation of gamma oscillations by acetylcholine. In contrast, we demonstrate a direct mechanism for the modulation of gamma oscillations where theta oscillation power and frequency remain constant.

We found that carbachol modulates the power of gamma oscillations in a bidirectional manner which initially suggested two distinct modulatory mechanisms mediated by multiple cholinergic receptors with different efficacies. Two potential mechanisms are an increase in excitability caused by activation of M1/M3 mAChRs and the decrease in inhibitory synaptic conductance caused by activation of M2 mAChRs or α4β2, α3β4 or α7 nicotinic receptors. Indeed, the power and frequency of persistent gamma oscillations generated by kainate have been shown to be modulated by nicotinic receptors (Wang *et al*., 2015). However, the frequency of gamma oscillations was unchanged by carbachol. This was surprising because acetylcholine has effects on both PV BC excitability and inhibitory synaptic transmission (Alkondon & Albuquerque, 2001; Ji *et al*., 2001; Szabo *et al*., 2010; Tang *et al*., 2011; Teles‐Grilo Ruivo & Mellor, 2013; Yi *et al*., 2014), which are key determinants of gamma oscillation frequency (Whittington *et al*., 1995; Wang & Buzsaki, 1996; Mann & Mody, 2010; Oren *et al*., 2010; Jadi & Sejnowski, 2014). Therefore, our data indicate a limited effect of carbachol on gamma oscillations via modulation of inhibition possibly due to a lower potency or efficacy at the receptor subtypes regulating inhibition (Jensen *et al*., 2003). In addition, our modelling predicts that networks with high excitatory‐to‐inhibitory synaptic input ratio will be resistant to modulations in gamma oscillation frequency (Fig. 7), whereas those with a lower ratio will be less resistant suggesting that different networks may vary in their frequency modulation. The lack of a role for M3 mAChRs, which might otherwise be expected to perform a similar role to M1 mAChRs, is supported by the differential functional expression and distribution of M3 and M1 mAChRs within the hippocampus (Porter *et al*., 2002; Dennis *et al*., 2015). Indeed, we found that the bidirectional modulation of gamma oscillations could be explained entirely by enhancing the excitability of PCs caused by activation of M1 mAChRs.

Optogenetic theta‐frequency stimulation of entorhinal cortex or hippocampus generates theta‐nested gamma oscillations that exhibit many important properties found in naturally occurring theta‐nested gamma including the relative timing of excitatory and inhibitory neurons within the network (Pastoll *et al*., 2013; Butler *et al*., 2016). In addition, as theta oscillation frequency and power may be kept constant, optogenetically stimulated theta‐nested gamma oscillations make an excellent model system to assess the modulation of gamma oscillations. Similarly, the mathematical model enabled us to probe the mechanism for cholinergic modulation of gamma oscillations by varying the known biophysical properties of cholinergic receptor subtype activation producing predictions which were subsequently tested experimentally revealing the key role for M1 mAChRs. Although the mathematical model was based on experimental biophysical parameters and recapitulated many of the core experimental observations, it did diverge in some respects to the experimental model most notably in the average gamma oscillation frequency which was lower in the mathematical model and may indicate potential differences between gamma oscillation mechanisms exhibited in the mathematical and experimental models. However, the predictions provided by the mathematical model were confirmed in the experimental model indicating the validity of the mathematical modelling approach.

Entrainment of neuronal activity to gamma oscillations is thought to be critical for local circuit computations and the transfer of information between brain regions (Colgin *et al*., 2009; Sohal *et al*., 2009; Ainsworth *et al*., 2012). The coherence and frequency of gamma oscillations are therefore critical for cognitive processing. Neuromodulators represent an excellent mechanism for modulating gamma oscillations and therefore regulating cognitive processing at a local and global level. However, the mechanisms by which neuromodulators modulate gamma oscillations are poorly understood. In this study we reveal that the neuromodulator acetylcholine modulates the power but not frequency of gamma oscillations in the hippocampus. This mechanism could work in tandem with the modulation of theta oscillations to mediate the effects of acetylcholine on cognition (Lee *et al*., 1994; McGaughy *et al*., 2000; Vandecasteele *et al*., 2014; Okada *et al*., 2015). Indeed optogenetically induced acetylcholine release enhances theta and gamma power in the hippocampus of anaesthetised mice, but reduces power in both bands in awake mice (Vandecasteele *et al*., 2014). Acetylcholine is released tonically in the hippocampus at high levels during performance on cognitively demanding tasks and to a lesser extent during REM sleep, but is generally very low during non‐REM sleep (or in the anaesthetised state) (Marrosu *et al*., 1995; Teles‐Grilo Ruivo *et al*., 2017). These observations suggest that gamma oscillations may be regulated in a bidirectional manner dependent on behavioural state.

Disruption to gamma oscillations is a prominent feature of schizophrenia and Alzheimer's disease. Cognitive control processes correlate with modulation of gamma oscillations in healthy humans, but this modulation is absent in schizophrenia (Cho *et al*., 2006). The disruption to gamma oscillations in schizophrenia is believed to result from a reduction in PV BC numbers and connectivity (Gonzalez‐Burgos *et al*., 2015). In parallel, disruptions to gamma oscillations in Alzheimer's disease are also thought to follow from a deficit in PV BCs and reversing these PV BC deficits in animal models reduces the associated cognitive deficits (Verret *et al*., 2012). An alternative strategy for cognitive enhancement could involve modulation of gamma oscillations by agents that target neuromodulator systems. In this study we identify one such target as the M1 mAChR. Interestingly, M1 agonists can enhance cognition in animals and human models of cognitive deficit (Bodick *et al*., 1997; Shirey *et al*., 2009; Digby *et al*., 2012; Nathan *et al*., 2013) supporting M1 mAChRs as a potential therapeutic target. Furthermore, our data suggest that the effects of any M1 mAChR agonist will be strongly dose dependent. This and the high M1 mAChR reserve in the hippocampus (Porter *et al*., 2002) will be important factors for the future development of M1 agonists as cognitive enhancers.

## Conflict of interest

The authors declare no competing financial interests.

## Author contributions

RTB, KT‐A and JRM designed the study; RTB performed experiments and modelling and analysed the data; LMB provided reagents; KT‐A and JRM supervised the project; RTB, LMB, KT‐A and JRM wrote the manuscript.

## Data accessibility

All primary data are archived at the University of Bristol and available on request.
