# Supplementary material for: Acetylcholine modulates gamma frequency oscillations in the hippocampus by activation of muscarinic M1 receptors
Source: Eur J Neurosci. 2017 May 8;45(12):1570–85. doi: 10.1111/ejn.13582 (PMC5518221; doi:10.1111/ejn.13582)
Supplement: Supplementary file 1 [file EJN-45-1570-s001.pdf]

Supplementary Figure 1

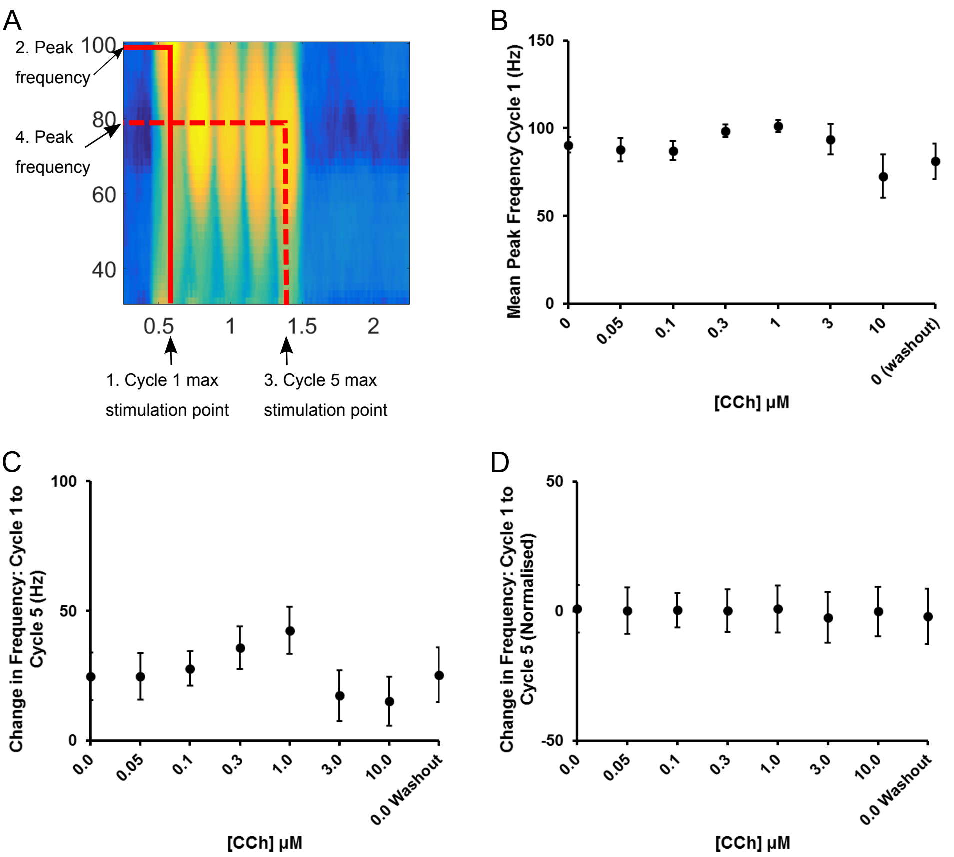

Supplementary Figure 1. Carbachol does not alter peak frequency or attenuation over 5 theta cycles. A) Spectrogram illustrating the measurement of peak frequency on the first and fifth theta cycles. B) Peak gamma frequency on cycle 1 of the theta stimulation was not affected by carbach ol (CCh). The attenuation in gamma frequency between the first and fifth theta cycles was not affected by carbachol when measured by the absolute frequency (C) or when normalised to baseline with no carbachol present (D). (ANOVA non-significant, n=11 slices from 9 animals)

Supplementary Figure 2

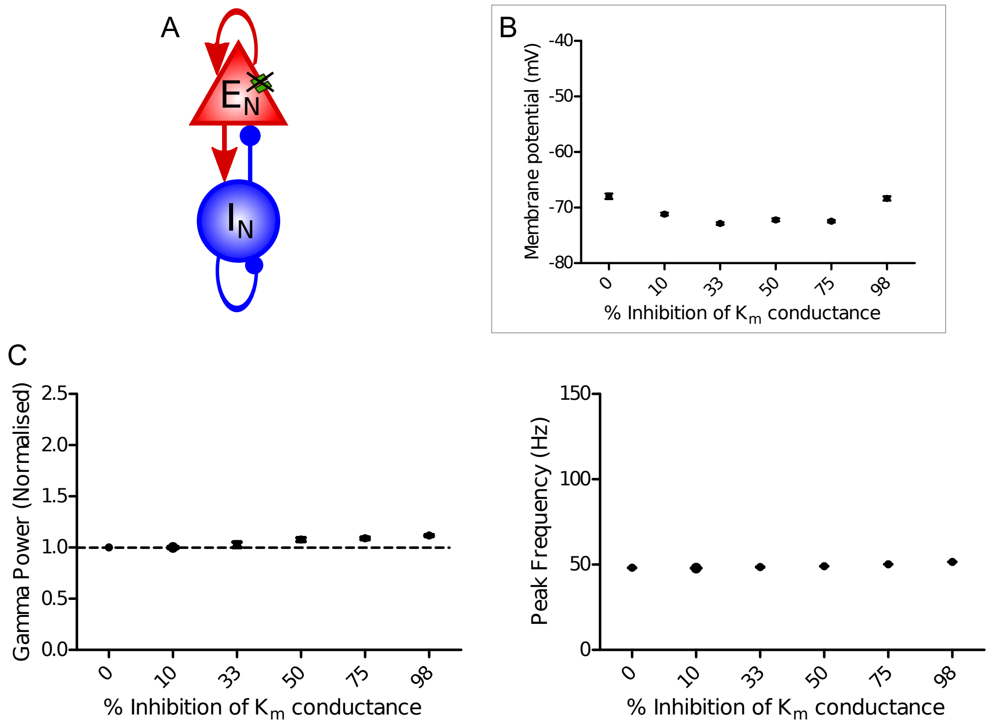

Supplementary Figure 2. M-curent inhibition does not replicate the effects of acetylcholine on gamma oscillations.

A) The effect of M1 mAChR activation was modelled by reducing Km conductance in the mathematical model. Inhibition of Km does not depolarise pyramidal neurons (B) or change gamma oscillation power or frequency (C).
